# Characteristics and Spectrum of Cardiotoxicity Induced by Various Antipsychotics: A Real-World Study From 2015 to 2020 Based on FAERS

**DOI:** 10.3389/fphar.2021.815151

**Published:** 2022-02-04

**Authors:** Luyao He, Yimin Yu, Yumei Wei, Jingjing Huang, Yifeng Shen, Huafang Li

**Affiliations:** Department of Shanghai Mental Health Center, School of Medicine, Shanghai Jiao Tong University, Shanghai, China

**Keywords:** antipsychotics, cardiotoxicity, cardiac adverse event, FAERS, reporting odds ratio (ROR), information component, spectrum

## Abstract

**Objective:** This study aimed to investigate the characteristics and spectrum of cardiotoxicity induced by various antipsychotics based on the United States Food and Drug Administration (FDA) Adverse Event Reporting System (FAERS) database.

**Methods:** Data of the FAERS database from the first quarter of 2015 to the fourth quarter of 2020 were downloaded for disproportionality analysis. The significant signal was evaluated by reporting odds ratios and information components with statistical shrinkage transformation.

**Results:** A total of 2,361,487 records were extracted for disproportionality analysis. Among the 10 antipsychotics, clozapine and amisulpride performed strong cardiotoxicity. Cardiomyopathy, cardiac arrhythmia, and Torsade de pointes/QT prolongation were the common cardiac adverse event induced by antipsychotics. Different characteristics of the spectrum of cardiotoxicity in various APs were discovered after further data mining. Moreover, evidence of the association between antipsychotics and eosinophilic myocarditis, peripartum cardiomyopathy was provided in this study.

**Conclusion:** Antipsychotics presented cardiotoxicity in different degrees, and more cardiac examinations should be monitored in patients with antipsychotics.

## Introduction

Schizophrenia is a chronic disease with a high lifetime prevalence of around 1% (Lieberman and First, 2018). Since schizophrenia patients need to take antipsychotics (APs) for a long time, which is the major treatment of schizophrenia (Marder and Cannon, 2019), drug safety is important for consideration when selecting APs. Meta-analyses of comparing efficacy and tolerability of antipsychotics displayed that various adverse events occurred in patients with APs, such as gaining weight, increasing prolactin, and prolonging QTc (Huhn et al., 2019).

It was reported that schizophrenia patients have a higher risk of cardiac disease and higher mortality of cardiac disease (Howell et al., 2019). The reason was complicated, perhaps relating to the disease itself or poor lifestyle habits common in schizophrenia patients. However, what is confirmed is that APs play an important role, as they have potential adverse effects on the heart.

There was much evidence supporting the association between APs and cardiac adverse events. Clozapine is the only antipsychotic authorized for treatment-resistant schizophrenia (Siskind et al., 2017). A significant number of patients discontinue clozapine due to cardiac adverse effects, in which drug-induced myocarditis is the most frequently reported (Patel et al., 2019a). Ziprasidone and amisulpride have a high risk of QTc prolongation, a type of arrhythmia that can lead to cardiac sudden death, according to a meta-analysis from Lancet (Huhn et al., 2019). Moreover, a tendency of gained weight and high blood glucose induced by using atypical antipsychotics potentially increased the risk of cardiovascular disease. Clinicians usually identified the cardiac adverse events induced by APs based on known associations provided by studies. Discontinuing APs promptly may prevent patients from developing fatal cardiac diseases. However, evidence of association was insufficient to clinical reference. The association between APs and rare cardiac adverse events which were only performed in case reports was still unclear.

Although the incidence of AP-induced cardiotoxicity is low, such as clozapine-induced myocarditis, which was reported at 0.2%–3% (Cook et al., 2015), and AP-induced QTc prolongation was estimated from 2% to 12% (Tisdale, 2016), the consequence could be serious when it occurred. It was difficult to investigate the association between APs and cardiotoxicity systematically and comprehensively in one cohort study or cross-sectional studies with a small sample.

The US Food and Drug Administration’s (FDA) Adverse Event Reporting System (FAERS) database supports data analysis of safety signals by its over 9 million spontaneous reports of potential adverse events. For now, many researchers explored associations between drugs and adverse events based on the FAERS database (Salem et al., 2018).

Our study, based on the FAERS database, aimed to detect the potential association between cardiotoxicity and APs, for the sake of increasing awareness of drug safety and promoting further research. The spectrum of cardiotoxicity also provided reference to clinicians and researchers investigating the AP-induced cardiotoxicity.

## Materials and Methods

### Data Source

The data of this study was extracted from adverse event reports recorded in the publicly available version of the United States Food and Drug Administration (FDA) Adverse Event Reporting System (FAERS) database (the first quarter of 2015 through the fourth quarter of 2020). The FAERS database collects spontaneous adverse event reports from healthcare professionals, patients, and pharmaceutical companies.

## Processing

The data set of FAERS contains seven data tables, which were named “DEMO,” “DRUG,” “REAC,” “OUTC,” “RPSR,” “THER,” and “INDI.” We used three tables (“DEMO,” “DRUG,” “REAC”) for analysis. “DEMO” displays information of demographic characteristics—patient’s age, sex, report country, event date, and so on. “DRUG” records details of medication. “REAC” lists the adverse event, and every adverse event is identified by preferred terms (PTs), as coded by the Medical Dictionary for Regulatory Activities (MedDRA). The data sets linked to each other by “primaryid,” which is a string of numbers generated by a system for identifying individual reports.

In order to increase the accuracy of this study, data were cleaned by excluding duplicates and reports without age or sex. Duplicate reports were considered as having the same key information such as age, sex, suspect drug, and adverse event reaction. We screened available Standardized MedDRA Queries (SMQs) by a “narrow” version in order to investigate the association between antipsychotics and cardiotoxicity adverse events. “Cardiac disease,” “Cardiomyopathy,” “Ischemic heart disease,” “Torsade de pointes/QT Prolongation,” and “Cardiac arrhythmia” were the four SMQs we selected to assess the cardiotoxicity.

For this study, we only considered the antipsychotics (APs) commonly used in China, including aripiprazole, amisulpride, clozapine, chlorpromazine, haloperidol, olanzapine, paliperidone, quetiapine, risperidone, and ziprasidone. The “prod_ai”—a variable in the FAERS database—was used to select the reports that related to target drugs in the database by searching the generic names. Reports that antipsychotics were recorded as suspected, interacting, or concomitant were included in the analysis.

## Data Analysis

The characteristics were described by median and interquartile ranges for measurement data, and numbers and percentages for enumeration data. Descriptive analyses were performed of cases and non-cases exposed to APs. Cases were defined reports with the target combination of drug and adverse events, and once reports with any SMQ were deemed to be cases, reports with other SMQs were considered as non-cases.

Reporting odds ratio (ROR), Bayesian confidence propagation neural networks of information components (IC), and both with the corresponding 95% confidence interval (CI) were calculated for performing disproportionality between cases and non-cases. Statistical shrinkage transformation was applied to obtain robust results (Norén et al., 2013). The calculation formulas are as follows:

$$ROR=\frac{N_{observed}+0.5}{N_{expected}+0.5}$$

$$IC=log_{2}\frac{N_{observed}+0.5}{N_{expected}+0.5}$$

$$N_{expected}=\frac{N_{drug}*N_{event}}{N_{total}}$$

$$ROR95\%CI=e^{\text{ln}\left(ROR\right)\pm 1.96\sqrt{\frac{1}{a}+\frac{1}{b}+\frac{1}{c}+\frac{1}{d}}}$$

$$IC_{025}=IC-3.3{\left(N_{observed}+0.5\right)}^{-0.5}-2{\left(N_{observed}+0.5\right)}^{-1.5}$$

$$IC_{975}=IC+2.4{\left(N_{observed}+0.5\right)}^{-0.5}-0.5{\left(N_{observed}+0.5\right)}^{-1.5}$$

where N_observed_ is the observed number of cases of the chosen combination of drug and adverse events, N_expected_ is the expected number of cases of the target combination, of drug and adverse events, N_drug_ is the total number of records of the chosen drug, and N_event_ is the total number of selected adverse events. N_total_ is the total number of records in the FAERS database during the period.

ROR and IC were calculated by two-by-two contingency tables for performing disproportionality between cases and non-cases. For better calibration of the results, the significant signal was considered when ROR_025_ was greater than 1 with at least 3 target combinations of drug and adverse event records and IC_025_ was greater than 0.

Both ROR and IC are figured up by comparing the total database, while IC_025_ is calculated in the cardiotoxicity spectrum of the significant signal, for representing the signal intensity. R version 4.1.0 was used for all analyses.

## Results

### Descriptive analysis

A total of 2,361,487 records were extracted, after removing the duplicate records and excluding the records without age and sex. The medium age of total patients included was 60 years (interquartile ranges: 45–71 years), of which cardiac failure was 66 years (interquartile ranges: 54–76 years), cardiomyopathy was 59 years (interquartile ranges: 45–70 years), ischemic heart disease was 63 years (interquartile ranges: 54–72 years), Torsade de pointes/QT prolongation was 59 years (interquartile ranges: 42–72 years), and cardiac arrhythmia was 63 years (interquartile ranges: 49–73 years). The number of adverse event records of females (58.4%) was more than that of males (41.6%) among the total number of patients. Females have a high proportion of records in cardiac failure (52.3%), cardiomyopathy (53.2%), Torsade de pointes/QT prolongation (53.8%), and cardiac arrhythmia (53.1%) and a low proportion in ischemic heart disease (42.3%). The specific details are shown in Table 1.

**TABLE 1:** Characteristics of cardiac adverse events and antipsychotics in FAERS database.

	*N*	Male	Female	Age
Total	23,611,487	973,648 (41.6%)	1,387,839 (58.4%)	60 (45–71)
Cardiac failure	48,210	22,973 (47.7%)	25,237 (52.3%)	66 (54–76)
Cardiomyopathy	9,077	4,244 (46.8%)	4,833 (53.2%)	59 (45–70)
Ischemic heart disease	50,477	29,149 (57.7%)	21,328 (42.3%)	63 (54–72)
TdP/QT Prolongation	12,818	5,918 (46.2%)	6,900 (53.8%)	59 (42–72)
Cardiac arrhythmia	24,198	11,346 (46.9%)	12,852 (53.1%)	63 (49–73)
AMI	1940	968 (49.9%)	972 (50.1%)	47 (33–58)
ARI	25,397	10,330 (40.7%)	15,067 (59.3%)	43 (29–57)
CHL	2,733	1,499 (54.8%)	1,234 (45.2%)	48 (34–62)
CLO	13,923	8,218 (59.0%)	5,705 (41.0%)	48 (34–61)
HAL	10,967	5,860 (53.4%)	5,107 (46.6%)	52 (34–67)
OLA	21,748	11,007 (50.6%)	10,741 (49.4%)	49 (33–62)
PAL	7,277	4,593 (63.1%)	2,684 (36.9%)	36 (24–51)
QUE	40,850	17,261 (42.3%)	23,589 (57.7%)	53 (37–68)
RIS	25,243	15,082 (59.7%)	10,161 (40.3%)	42 (23–62)
ZIP	3,349	1,212 (36.2%)	2,137 (63.8%)	43 (29–56)

AMI, amisulpride; ARI, aripiprazole; CHL, chlorpromazine; CLO, clozapine; HAL, haloperidol; OLA, olanzapine; PAL, paliperidone; QUE, quetiapine; RIS, risperidone; ZIP, ziprasidone.

In 2,361,487 reports, only 810,327 (34.31%) reports have details about country, of which the top ten were United States (452,695, 55.87%), Canada (57,010, 7.04%), France (46,758, 5.77%), Japan (35,535, 4.39%) Great Britain (32,295, 3.99%), Germany (27,117, 3.35%), Italy (20,346, 2.51%), Spain (11,003, 1.36%), Brazil (8,894, 1.10%), and China (8,359, 1.03%). In the 121,285 reports with cardiotoxicity, only 48,527 (40.01%) reports record the country where adverse events occurred, of which the top ten were United States (27,316, 56.29%), Canada (3,400, 7.00%), Japan (2,344, 4.83%), France (2,304, 4.75%), Germany (1896, 3.91%), Great Britain (1,523, 3.14%), Italy (929, 1.91%), Brazil (645, 1.33%), Spain (581, 1.20%), and China (475, 0.98%).

**FIGURE 1:**
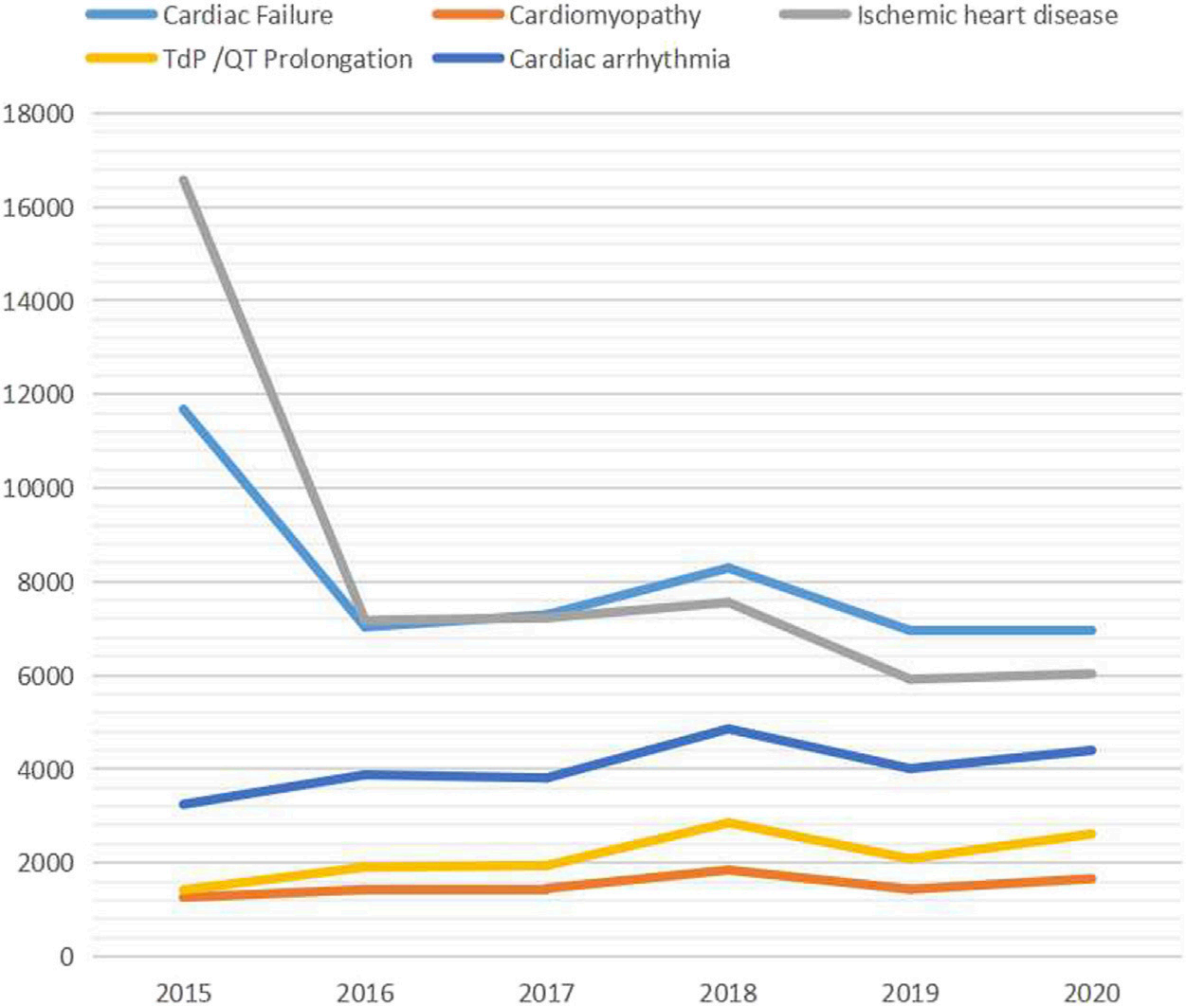
The time trend of cardiac adverse events.

Figure 1 shows the time trend of cardiac adverse events. The reports of cardiac failure were most in 2015 (*N* = 11,677) and decreased during 2016–2020 (2016: *N* = 7,033, 2017: *N* = 7,292, 2018: *N* = 8,291, 2019: *N* = 6,957, 2020: *N* = 6,960). The tendency was similar with ischemic heart disease, which reported most in 2015 (*N* = 16,572) and dropped in 2016 (*N* = 7,176), 2017 (*N* = 7,226), 2018 (*N* = 7,555), 2019 (*N* = 5,916), and 2020 (*N* = 6,032). There was an upward tendency of reports of cardiomyopathy (2015: *N* = 1,260, 2016: *N* = 1,426, 2017: *N* = 1,453, 2018: *N* = 1844, 2019: *N* = 1,434, 2020: *N* = 1,660), TdP/QT prolongation (2015: *N* = 1,420, 2016: *N* = 1905, 2017: *N* = 1940, 2018: *N* = 2,853, 2019: *N* = 2085, 2020: *N* = 2,615), and cardiac arrhythmia (2015: *N* = 3,246, 2016: *N* = 3,878, 2017: *N* = 3,810, 2018: *N* = 4,857, 2019: *N* = 4,009, 2020: *N* = 4,398) respectively.

## Signal values associated with different cardiotoxicities

**FIGURE 2:**
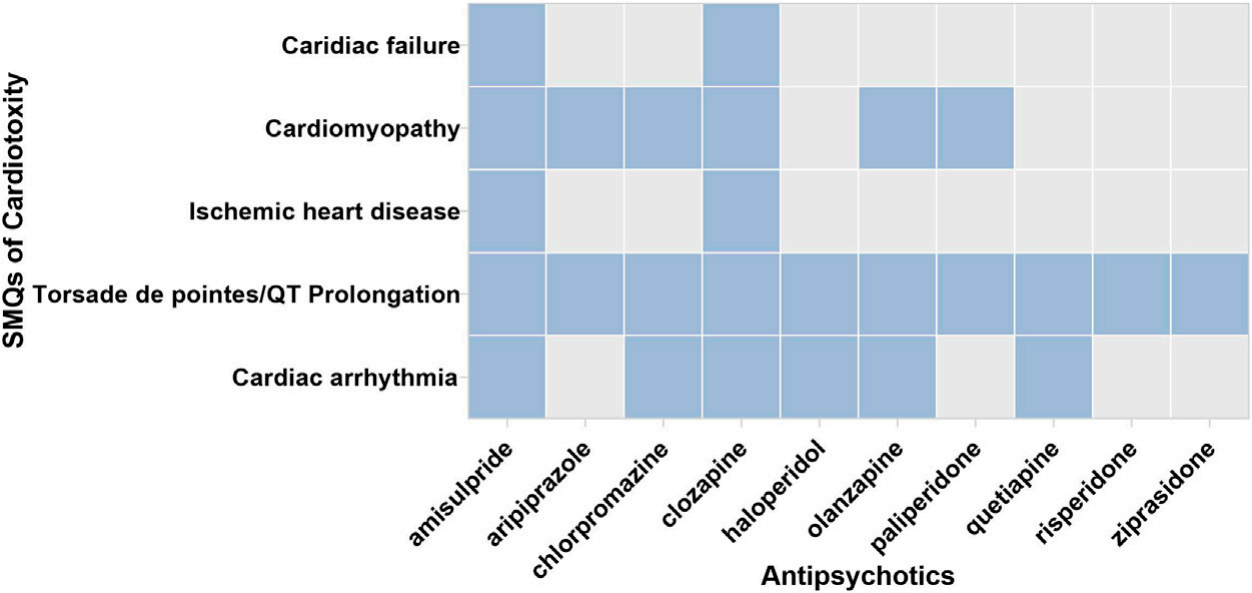
The associations between APs and respective SMQs. SMQ: the blue blocks represent the significant associations between APs and SMQs; the gray blocks represent the nonsignificant association between APs and SMQs.

Figure 2 displays the association between APs and selected Standardized MedDRA Queries (SMQs). Most APs were observed with a higher reporting frequency in cardiomyopathy, Torsade de pointes/QT prolongation, and cardiac arrhythmia compared with the whole database. Among ten APs, amisulpride and clozapine showed disproportionality in all five SMQs. The specific signal value is displayed in Table 2.

**TABLE 2:** The association of cardiotoxicities with antipsychotics.

Cardiac failure						
Drugs	ROR	ROR_025_	ROR_975_	IC	IC_025_	IC_975_
AMI	1.533	1.483	1.585	0.617	0.192	0.922
ARI	0.539	0.535	0.542	−0.893	−1.091	−0.749
CHL	0.950	0.915	0.987	−0.073	−0.530	0.253
CLO	1.189	1.182	1.196	0.250	0.070	0.380
HAL	1.125	1.116	1.134	0.170	−0.040	0.321
OLA	0.906	0.901	0.910	−0.143	−0.308	−0.024
PLA	0.399	0.386	0.413	−1.325	−1.757	−1.015
QUE	0.746	0.744	0.748	−0.423	−0.555	−0.327
RIS	0.635	0.631	0.639	−0.655	−0.838	−0.523
ZIP	0.327	0.299	0.357	−1.614	−2.328	−1.113
**Cardiomyopathy**						
**Drugs**	**ROR**	**ROR**_**025**_	**ROR_975_**	**IC**	**IC_025_**	**IC_975_**
AMI	5.216	4.966	5.478	2.383	1.863	2.754
ARI	1.483	1.463	1.503	0.568	0.294	0.767
CHL	3.226	3.047	3.415	1.690	1.126	2.090
CLO	5.063	5.025	5.102	2.340	2.140	2.485
HAL	1.348	1.302	1.396	0.431	−0.009	0.746
OLA	2.230	2.206	2.254	1.157	0.915	1.332
PAL	1.528	1.459	1.600	0.612	0.104	0.974
QUE	1.178	1.165	1.191	0.236	−0.007	0.412
RIS	0.733	0.713	0.754	−0.448	−0.841	−0.165
ZIP	1.234	1.090	1.396	0.303	−0.539	0.887
**Ischemic heart disease**						
**Drugs**	**ROR**	**ROR**_**025**_	**ROR_975_**	**IC**	**IC_025_**	**IC_975_**
AMI	1.489	1.441	1.539	0.575	0.153	0.877
ARI	0.671	0.667	0.675	−0.576	−0.579	−0.371
CHL	0.789	0.756	0.824	−0.341	−0.832	0.009
CLO	1.749	1.742	1.756	0.807	0.662	0.912
HAL	0.764	0.756	0.773	−0.388	−0.635	−0.209
OLA	0.676	0.672	0.680	−0.565	−0.752	−0.430
PLA	0.580	0.567	0.593	−0.786	−1.135	−0.534
QUE	0.724	0.722	0.726	−0.466	−0.597	−0.371
RIS	0.460	0.465	0.464	−1.120	−1.330	−0.968
ZIP	0.354	0.327	0.383	−1.499	−2.168	−1.028
**Torsade de pointes/QT prolongation**						
**Drugs**	**ROR**	**ROR**_**025**_	**ROR_975_**	**IC**	**IC_025_**	**IC_975_**
AMI	8.749	8.561	8.940	3.129	2.791	3.373
ARI	3.061	3.046	3.076	1.614	1.453	1.731
CHL	5.902	5.764	6.037	2.561	2.212	2.813
CLO	4.410	4.383	4.437	2.141	1.960	2.272
HAL	4.156	4.122	4.190	2.055	1.846	2.207
OLA	5.040	5.022	5.058	2.333	2.198	2.432
PLA	1.588	1.538	1.638	0.667	0.249	0.967
QUE	3.544	3.534	3.553	1.825	1.708	1.911
RIS	2.520	2.505	2.535	1.333	1.156	1.462
ZIP	2.971	2.865	3.081	1.571	1.123	1.892
**Cardiac arrhythmia**						
**Drugs**	**ROR**	**ROR**_**025**_	**ROR_975_**	**IC**	**IC_025_**	**IC_975_**
AMI	2.036	1.939	2.138	1.026	0.506	1.397
ARI	1.118	1.110	1.126	0.161	−0.033	0.301
CHL	1.772	1.702	1.844	0.825	0.355	1.161
CLO	1.359	1.345	1.373	0.442	0.205	0.614
HAL	1.829	1.812	1.847	0.871	0.641	1.038
OLA	1.565	1.556	1.574	0.646	0.469	0.774
PLA	0.873	0.846	0.900	-0.197	−0.608	0.099
QUE	1.524	1.519	1.528	0.607	0.477	0.702
RIS	1.136	1.129	1.144	0.184	−0.008	0.324
ZIP	1.393	1.393	1.452	0.478	−0.002	0.821

AMI, amisulpride; ARI, aripiprazole; CHL, chlorpromazine; CLO, clozapine; HAL, haloperidol; OLA, olanzapine; PAL, paliperidone; QUE, quetiapine; RIS, risperidone; ZIP, ziprasidone.

Focusing on cardiac failure, APs excluding amisulpride (ROR_025_ = 1.508, IC_025_ = 0.192) and clozapine (ROR_025_ = 1.185, IC_025_ = 0.070) were not observed to have a significant signal. Focusing on cardiomyopathy, over half of APs have a significant signal—amisulpride (ROR_025_ = 5.089, IC_025_ = 1.863), aripiprazole (ROR_025_ = 1.473, IC_025_ = 0.294), chlorpromazine (ROR_025_ = 3.135, IC_025_ = 1.126), clozapine (ROR_025_ = 5.044, IC_025_ = 2.140), olanzapine (ROR_025_ = 2.218, IC_025_ = 0.915), and paliperidone (ROR_025_ = 1.493, IC_025_ = 0.104). Focusing on ischemic heart disease, only amisulpride (ROR_025_ = 1.465, IC_025_ = 0.153) and clozapine (ROR_025_ = 1.746, IC_025_ = 0.662) have a significant signal. Focusing on Torsade de pointes/QT prolongation, all APs (amisulpride: ROR_025_ = 8.561, IC_025_ = 2.791; aripiprazole: ROR_025_ = 3.046, IC_025_ = 1.453; chlorpromazine: ROR_025_ = 5.770, IC_025_ = 2.212; clozapine: ROR_025_ = 4.383, IC_025_ = 1.960; haloperidol: ROR_025_ = 4.122, IC_025_ = 1.846; olanzapine: ROR_025_ = 4.383, IC_025_ = 1.960; quetiapine: ROR_025_ = 3.534, IC_025_ = 1.708; risperidone: ROR_025_ = 2.505, IC_025_ = 1.156; ziprasidone: ROR_025_ = 2.865, IC_025_ = 1.123) were observed to have a significant value, among which amisulpride showed the strongest signal (ROR = 8.749, IC = 3.129). Focusing on cardiac arrhythmia, a significant association was demonstrated in six APs, including amisulpride (ROR_025_ = 1.987, IC_025_ = 0.506), chlorpromazine (ROR_025_ = 1.737, IC_025_ = 0.355), clozapine (ROR_025_ = 1.352, IC_025_ = 0.205), haloperidol (ROR_025_ = 1.820, IC_025_ = 0.641), olanzapine (ROR_025_ = 1.560, IC_025_ = 0.469), and quetiapine (ROR_025_ = 1.521, IC_025_ = 0.477).

## The spectrum of cardiotoxicity varies in APs

**FIGURE 3:**
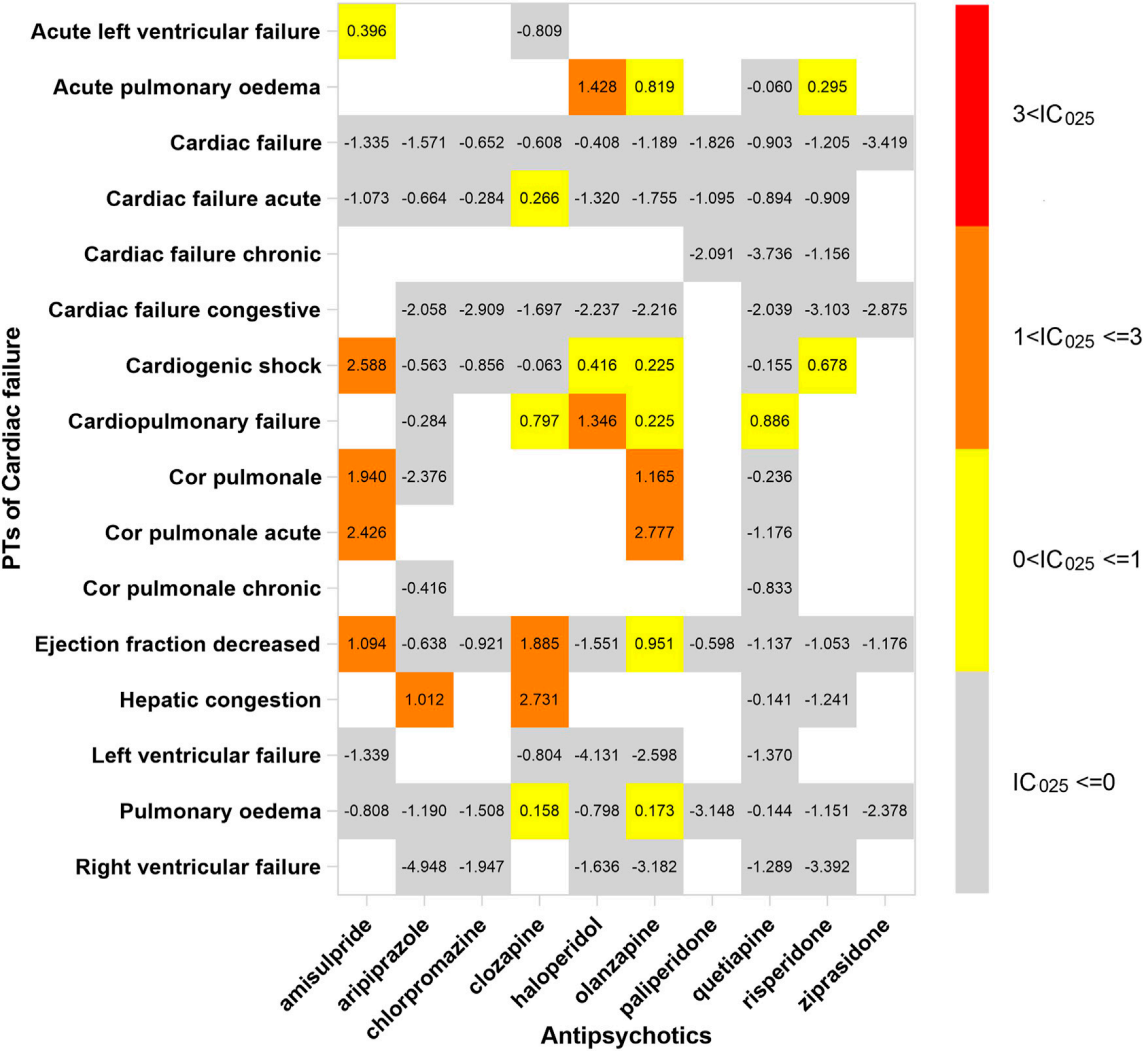
The spectrum of cardiac failure induced by various APs. PT: preferred term; IC_025_: the lower end of the 95% confidence interval of the information component. The numbers in blocks represent the value of IC_025_ of each combination of target antipsychotic and PT. Ejection fraction decreased is the common PT both in cardiac failure and in cardiomyopathy.

**FIGURE 4:**
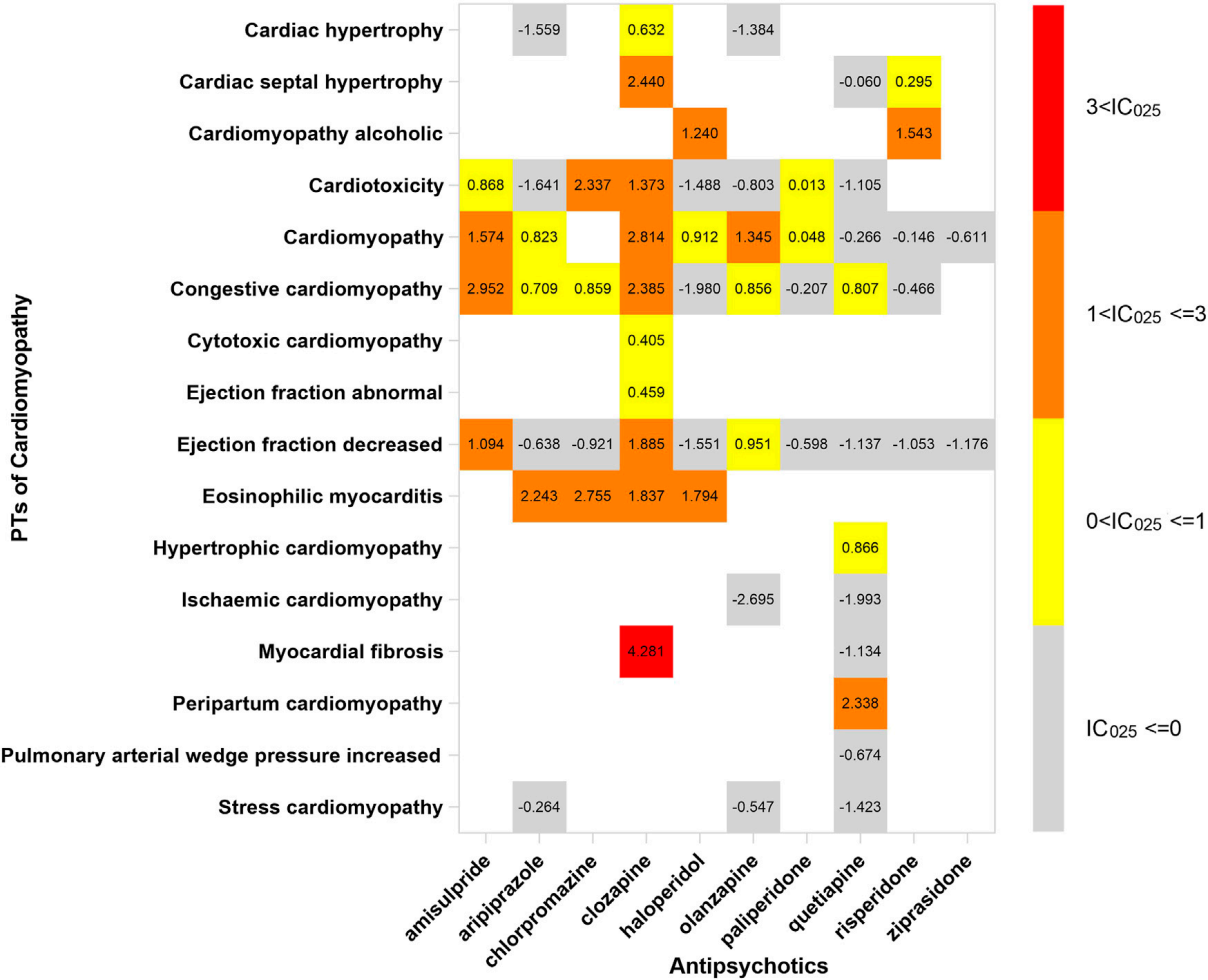
The spectrum of cardiomyopathy induced by various APs. PT: preferred term; IC025: the lower end of the 95% confidence interval of the information component. The numbers in blocks represent the value of IC025 of each combination of target antipsychotic and PT. The ejection fraction decreased is the common PT both in cardiac failure and in cardiomyopathy.

**FIGURE 5:**
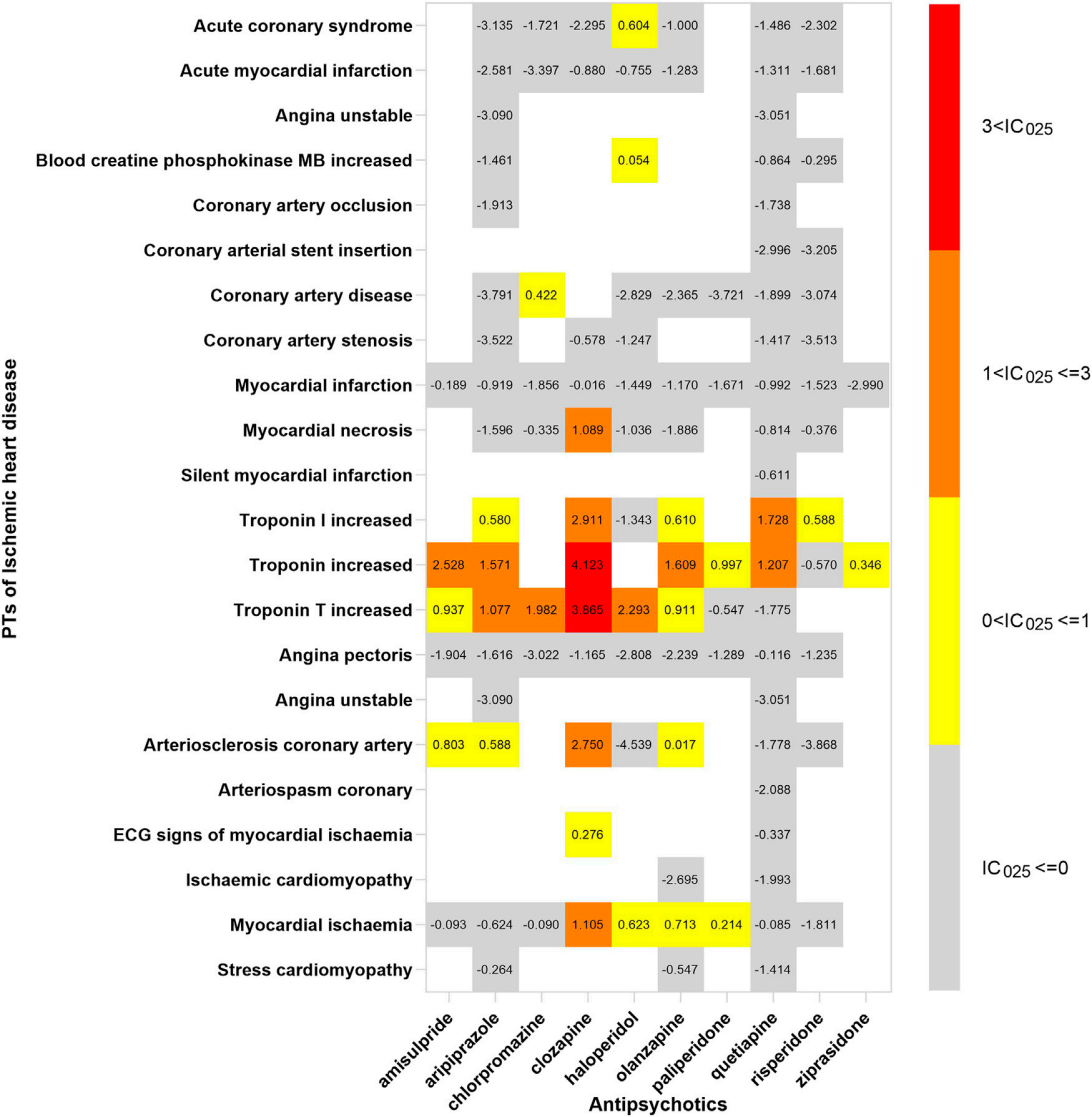
The spectrum of ischemic heart disease induced by various APs. PT: preferred term; IC_025_: the lower end of the 95% confidence interval of the information component. The numbers in blocks represent the value of IC_025_ of each combination of target antipsychotic and PT.

**FIGURE 6:**
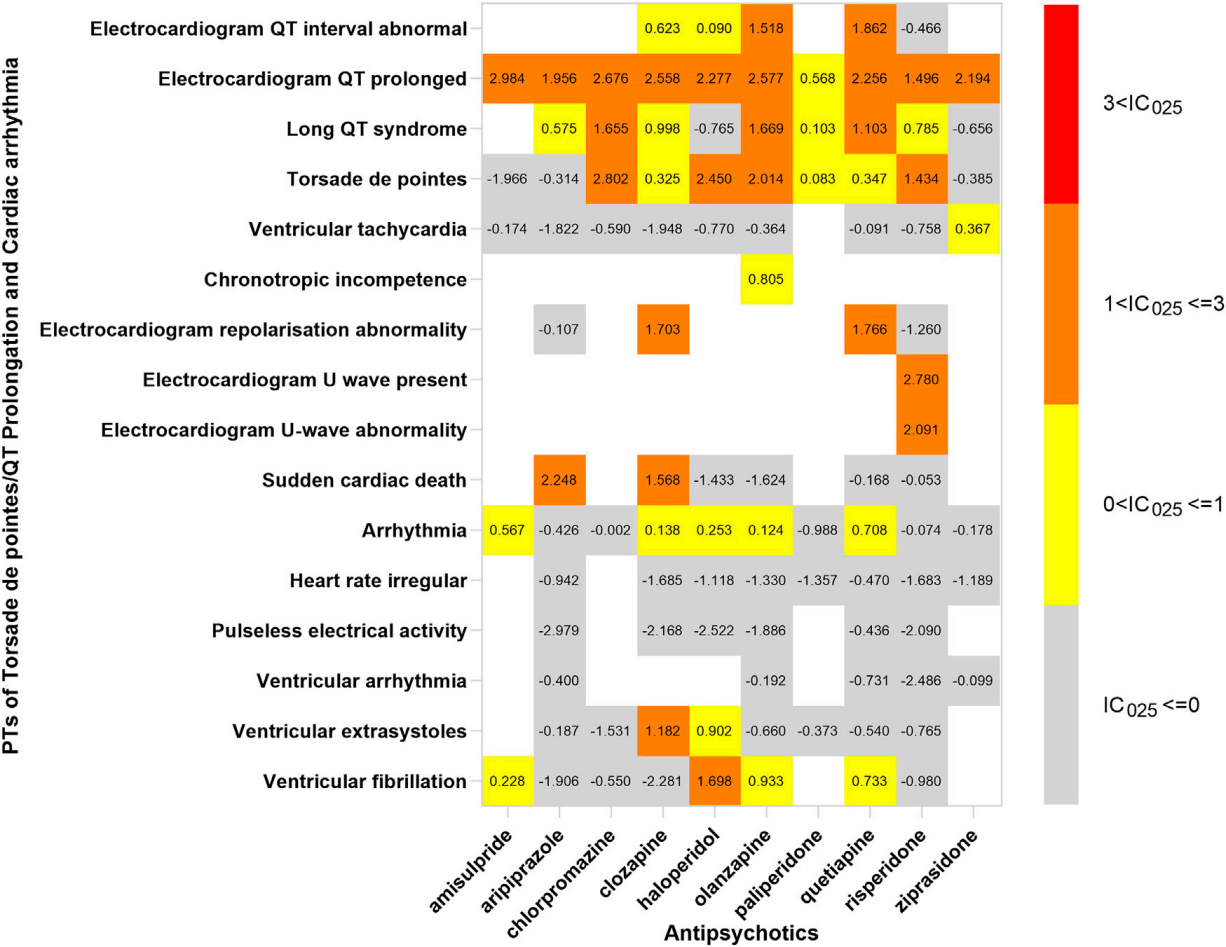
The spectrum of Torsade de pointes/QT prolongation and cardiac arrhythmia induced by various APs. PT: preferred term; IC_025_: the lower end of the 95% confidence interval of the information component. The numbers in blocks represent the value of IC_025_ of each combination of target antipsychotic and PT. Torsade de pointes and ventricular tachycardia are the common PTs both in Torsade de pointes/QT prolongation and cardiac arrhythmia.

In order to investigate the association between various APs and specific cardiac adverse events, we explored the spectrum of cardiotoxicity of each; details are shown in Figures 3–6. Clozapine showed the widest spectrum of cardiac adverse events with 28 PTs as the detected signal, ranging from IC_025_ = 0.138 (arrhythmia) to IC_025_ = 4.281 (myocardial fibrosis). The second one was olanzapine with 20 PTs as the detected signal, ranging from IC_025_ = 0.017 (arteriosclerosis coronary artery) to IC_025_ = 2.777 (cor pulmonale acute). It was worth noting that their (clozapine and olanzapine) distribution of the spectrum of cardiac adverse events was quite different. Compared to olanzapine, clozapine seemed to be more aggressive to myocardial cells, because most of the signal was related to cardiomyopathy. For olanzapine, the signal was commonly related to cardiac failure. Three overlapping PTs—troponin increased, troponin I increased, and troponin T increased—were detected in clozapine and olanzapine.

Ziprasidone was with the narrowest PTs, of which cardiac hypertrophy (IC_025_ = 2.508) was the strongest signal, and the rest were electrocardiogram QT prolonged (IC_025_ = 2.194), ventricular tachycardia (IC_025_ = 0.367), and troponin increase (IC_025_ = 0.346). A total of 14 signals were detected in quetiapine, and the strongest was peripartum cardiomyopathy (IC_025_ = 2.338); most of the signals were concentrated to cardiac arrhythmia. The strongest signal observed in chlorpromazine was Torsade de pointes (IC_025_ = 2.802), a life-threatening cardiac arrhythmia. Coincidently, the same was in haloperidol (IC_025_ = 2.450).

Electrocardiogram QT prolonged was the only potential signal detected in all 10 APs. Besides, Torsade de pointes and troponin T increased were two signals detected in 6 APs.

## Discussion

APs are widely used for psychiatric disorders, especially schizophrenia. People were concerned about the adverse events induced by APs, since they impact the drug selection in the long-term treatment. In the cardiac system, the association between APs and cardiac adverse events has emerged, as the number of reports increased (Ray et al., 2009; Correll and Nielsen, 2010). A comprehensive realization of cardiotoxicity induced by APs may help decrease the risk of cardiac death and give references to clinicians. However, it is difficult to investigate the association systematically on account of the rare occurrence of cardiac adverse events. We conducted a pharmacovigilance study by using the FAERS database and provide more characteristics on the cardiotoxicity induced by APs. The main result found in our study is that APs were all associated with cardiotoxicity, and the spectrum displaying the cardiotoxicities were different in some aspects. We also found the potential association between APs and some rare cardiac adverse events.

Compared with the entire database, all ten APs performed cardiotoxicity, especially clozapine and amisulpride, which were associated with all SMQs in our study. Clozapine was the first atypical antipsychotic drug, but it had been limited due to potentially serious adverse reactions, such as cardiotoxicity. As the systematic review concluded, cardiomyopathy and electrocardiographic (ECG) abnormalities were the major cardiac adverse event in clozapine (Curto et al., 2016; Patel et al., 2019b). Although the morbidity of cardiomyopathy induced by clozapine is very low, estimated one in a thousand (Longhi and Heres, 2017), the risk of death increased once it occurred. Discontinuation of clozapine is the key to recovering from cardiac disease induced by clozapine, so discerning the cardiac adverse events in early time is very important. We suggested that cardiac examination should be monitored in patients taking clozapine, especially those who have had a heart disease.

According to our study, there exists an association between cardiac failure and clozapine, although it was only supported by case reports (Thomas et al., 2006; Whiskey et al., 2020). The occurrence of cardiac failure potentially followed subclinical neuroleptic (antipsychotic) cardiomyopathy, such as dilated cardiomyopathies, which was characterized by congestive chronic heart failure (Volkov, 2018). This is why these signals—“ejection fraction decreased” and “hepatic congestion”—were detected in further data mining. The process of development of cardiac dysfunction was slow, and some biomarkers emerged before clinical symptoms. Brain natriuretic peptide (BNP) and N-terminal pro-B-type natriuretic peptide (NT-proBNP) were two hormones secreted by the myocardial cell against myocardial fibrosis and hypertrophy, and cardiomyopathy and cardiac failure can be ruled out if their level was normal. It is better to take natriuretic peptide detection when the patient was suspected to have cardiac adverse events induced by not only clozapine but also other APs.

We were of the opinion that caution should be exercised in associating clozapine and ischemic heart disease; although it was indicated in our study, the association was rarely reported. The significant association was probably due to the signal of “myocardial necrosis” and “troponin (including Troponin I and Troponin T) increased,” after further exploring. It is hard to determine if necrosis of the myocardium was totally contributing to cardiovascular disease, not cardiomyopathy. Although the strategy of our study limited specificity, it increased the sensitivity of signal detection. The association should be further verified in a prospective study in which the diagnosis of ischemic heart disease was employed by digital subtraction angiography.

Amisulpride shows the same cardiotoxicity as clozapine displayed in our study, particularly in cardiomyopathy and ischemic heart disease. Moreover, we thought that the mechanism was similar to clozapine, which we discussed above. For cardiac failure, there were subtle differences in cardiac failure of both APs, since we found that amisulpride tended to provoke acute cardiac dysfunction, such as acute left ventricular failure, cardiogenic shock, and acute cor pulmonale, after we compare the spectrum of cardiotoxicity. The underlying mechanism still awaits to be revealed.

In a double-blind placebo control trial, more cardiac adverse events occurred in amisulpride than placebo, especially arrhythmia (Barnes et al., 2018). It was in line with our result. In addition, amisulpride demonstrated the strongest signal in “Torsade de pointes/QT prolongation,” which was consistent with a meta-analysis (Huhn et al., 2019). In consideration of the strong cardiotoxicity of clozapine and amisulpride, the combination of these two APs was dangerous to patients with a high risk of cardiac disease.

Most of the cardiotoxicity of APs was performed in form of cardiomyopathy and cardiac arrhythmia. Electrocardiogram QT prolonged is the only signal in all ten drugs.

Our result was in keeping with an observational study, which recorded cardiac adverse events of 404,009 patients during 20 years and revealed that QTc prolongation, myocardial disease, and arrhythmia were the most common events (Friedrich et al., 2020). The association between APs and cardiomyopathy was supported by case reports and other studies (Muench and Hamer, 2010; Puttegowda et al., 2016; Smolders and Smolders, 2017). However, the mechanism of AP-induced cardiotoxicity was still inconclusive. Myocardial fibrosis is a characteristic feature in AP-induced cardiotoxicity and is already certified in schizophrenia patients by cardiac MRI (Pillinger et al., 2019). Recently, researchers found that the expression of three spliceosome signal proteins, related to cardiomyocyte adaptation and cardiac remodeling, was associated with myocardial fibrosis in rats with AP-induced cardiotoxicity by using proteomic and transcriptomic analyses (Wang et al., 2021). Moreover, it has been found that APs can increase myocyte apoptosis by various mechanisms, blocking the dopamine D2/3 receptors or bidirectional regulation of cannabinoid receptors (Li et al., 2014; Li et al., 2019). The mechanism discussed above demonstrated that the damage led by the toxic effect of APs concentrated on myocardial cells, not angiography. This may explain why cardiomyopathy and cardiac arrhythmia were the commonest cardiac adverse events in most APs.

Our study detected a strong signal in the combination of clozapine and myocardial fibrosis, and it provided evidence for myocardial fibrosis which was the main pathological manifestation of AP-induced cardiotoxicity. What is more, the signal of cardiac hypertrophy and cardiac septal hypertrophy detected in clozapine testified cardiomyocyte adaptation and cardiac remodeling in patients with APs. We did not find the association between myocardial fibrosis and the other APs, partly due to limitations of spontaneous reporting that not all adverse events in the database were verified by pathological examination. The other explanation could be the bias (reports of the association between clozapine and myocardial fibrosis increased the cases in the database) (Pariente et al., 2007).

Cardiac arrhythmia was another adverse event that was reported frequently. One explanation is that EGC was routinely monitored in patients, but the other cardiac examinations, such as ultrasonography and computerized tomography, were not considered routine examinations. Ventricular tachycardia and ventricular fibrillation were the common reasons for the sudden cardiac death in patients taking APs (Zhu et al., 2019). In a 6-year follow-up cohort study, SCD occurred in 2% of patients during the period. A nationwide case-crossover study indicated that the risk of ventricular arrhythmia increased 1.53-fold after using APs (Wu et al., 2015). Torsade de pointes was the most common form of cardiac arrhythmia induced by various APs. Moreover, disproportionality analysis showed that Torsade de pointes/QT prolongation was the only cardiac adverse event associated with all ten APs. Inconsistently, a study based on the FAERS database displayed that amisulpride and olanzapine were associated with TdP through a cumulative approach of the search strategy (Poluzzi et al., 2013). Our results might be inaccurate as we did not adjust variables that affected QT, such as age, sex, and dose. Moreover, it interfered with disproportionality analyses. After a detailed investigation of the spectrum of TdP/QT prolongation, we discovered that all APs were still associated with electrocardiogram QT prolonged, but not all with TdP. Actually, the association between most APs and TdP was unclear, excluding chlorpromazine, haloperidol, pimozide, and thioridazine which were the known antipsychotics inducing TdP (Tisdale, 2016). APs induced QT prolongation *via* inhibiting hERG K^+^ channels. Although our results needed to be replicated, it displayed a trend, in keeping with available data that most APs affected hERG K^+^ channels and associated with QT prolongation (Titier et al., 2005). The electrocardiogram should be monitored when using APs. Furthermore, olanzapine, which was considered as a low-risk AP, was significantly associated with TdP in our study and previous research based on FAERS (Poluzzi et al., 2013). Clinicians should be cautious of high-risk patients with olanzapine.

We found different characteristics of the spectrum of cardiotoxicity in various APs. Clozapine performed strong cardiotoxicity; most numbers of signals were detected in cardiomyopathy, ischemic heart disease, and cardiac arrhythmia. Although olanzapine was not associated with cardiac failure initially, we found 7 signals after exploring the spectrum of cardiac failure. Olanzapine was detected to have the most number of signals in cardiac failure, compared with the other APs. The signals of quetiapine and ziprasidone were mainly concentrated in Tdp/QT prolongation and cardiac arrhythmia. It was difficult to explain the difference in spectrum with what we know so far, but what we confirmed is that it originated from the complicated pharmacological effect of APs.

Given the lack of similar studies, we could not compare our results to others, but our study provides evidence for further studies. The different characteristics were beneficial for a precise treatment that may decrease the occurrence of the adverse event. For example, clozapine was suggested to be avoided in patients with a high risk of cardiomyopathy, and olanzapine was not the first option for patients who were potentially at risk of cardiac failure. Besides, antipsychotic polypharmacy (APP) was common in the treatment of mental illness, and the prevalence of APP was 19.6% globally and 34.2% in China (Gallego et al., 2012; Li et al., 2015). Although there was not enough evidence supporting that the risk would increase after using more than one AP, we still give the advice that the combination of quetiapine and ziprasidone was not recommended in patients who had cardiac arrhythmia before or with a high risk of TdP/QT prolongation. Meanwhile, the electrocardiogram should be monitored more frequently in patients with quetiapine or ziprasidone.

The association between APs and rare cardiac adverse events was observed in this study. Eosinophilic myocarditis is one type of myocarditis with a low incidence rate. It is usually revealed after postmortem examination, due to lack of typical clinical manifestations. Diffuse or focal myocardial inflammation is the main pathological feature. Eosinophils in the myocardium can cause damage to myocardial cells by releasing destructive proteins. Eosinophilic myocarditis can be caused by drugs with an allergic hypersensitivity reaction. Case series showed that eosinophilic myocarditis can be induced by APs (Sahpaz et al., 2016). Due to the limited cases and insufficient evidence, this fatal cardiac adverse event induced by APs did not get enough attention.

Clozapine, aripiprazole, chlorpromazine, and haloperidol were the 4 APs associated with eosinophilic myocarditis, according to our results. The association was rarely seen in psychiatric patients because an autopsy was not performed conventionally. Although evidence was insufficient due to the absence of prospective studies, the association still should be noticed. Actually, excluding the 4 APs mentioned above, olanzapine was reported of raising the eosinophil count (Tsamakis et al., 2021). Moreover, it was suspected of inducing eosinophilic myocarditis in case reports (Vang et al., 2016). The association between olanzapine or other APs and eosinophilic myocarditis was possibly concealed by the limitation of spontaneous reports, since autopsies were not performed in every case.

Peripartum cardiomyopathy (PPCM) is a life-threatening cardiac disease occurring during pregnancy. The patients performed left ventricular systolic dysfunction developed by vascular changes. We detected an association between quetiapine and PPCM from the spectrum of cardiomyopathy. Although the association was performed in the case report (Kaler et al., 2013), we thought the evidence was not sufficient. As it was surveyed, quetiapine was the most commonly AP used during pregnancy (Park et al., 2017). We cannot rule out the possibility that the signal of PPCM was potentially due to the high frequency of using quetiapine.

There is still much work to do for investigating the association between APs and these rare cardiac adverse events. Our study provided valuable evidence for clinical reference.

There are some limitations in the present study. FAERS is a spontaneous reporting system with typical weaknesses, such as subjective reports, missing data, and duplicate reports. Although we developed strategies to reduce the duplicates for increasing the accuracy of our results, the final dataset potentially contained the duplicates. Besides, underreporting was a common limitation of a spontaneous reporting system. Reports from different sources limited the verification of rationality of reports by laboratory tests, pathological examination, or even autopsy. Overreporting is another typical limitation, which may increase the occurrence of false positive results in analysis. The association between TdP and some drugs was overreported in other spontaneous reporting systems, such as the VigiBase and European pharmacovigilance databases (De Bruin et al., 2005; Grouthier et al., 2018). Variables such as weight, dose, and time to onset were not included in the study, so we cannot estimate the association between dose and risk. We did not consider the complex polypharmacy of APs and only take AP monotherapy into account. However, this study which was based on big data from the FAERS database explored the cardiotoxicity of APs systematically and discovered the characteristics and spectrum of cardiotoxicity induced by APs, which provided evidence for clinical practice.

## Conclusion

Our study conducted a comprehensive analysis and systematically valued and quantified the potential cardiotoxicity induced by APs, which displayed that ten APs in this study presented cardiotoxicity in different degrees and provided valuable evidence for clinical practice and medication monitoring. The characteristics of the spectrum of cardiotoxicity induced by various APs, discovered in this study, can help clinicians know the different cardiotoxicity of APs and guide clinical practice. Several cardiac adverse events have not gotten enough attention yet, and we suggested more cardiac examinations should be monitored in high-risk patients.

## Data Availability

The original contributions presented in the study are included in the article/Supplementary Material; further inquiries can be directed to the corresponding authors.
